# Evaluation of Fluoraphat Pro and VivaSens® In-Office Desensitizing Agents in Controlling Natural Dental Hypersensitivity: A Clinical Study

**DOI:** 10.7759/cureus.51463

**Published:** 2024-01-01

**Authors:** Nourah M Aljasser, Roula S Al-Bounni

**Affiliations:** 1 Restorative Dentistry Department, Riyadh Specialized Dental Centre, Ministry of Health, Riyadh, SAU; 2 Restorative Dentistry Department, College of Medicine and Dentistry, Riyadh Elm University, Riyadh, SAU

**Keywords:** risk, vivasens, fluoraphat pro, desensitizing agents, dentinal hypersensitivity

## Abstract

Introduction

Dental erosion from acidogenic diets, vigorous tooth brushing, excessive tooth whitening, gingival recession, periodontal debridement, or surgery may induce dentine hypersensitivity (DH). It manifests as a phenomenon observed in structurally intact teeth devoid of pathological or defective conditions. Hence, this study aimed to evaluate DH risk reduction after using Fluoraphat Pro (Neumunster, Germany) and VivaSens^®^ (Ivoclar Vivadent, Schaan, Liechtenstein, Switzerland) in-office desensitizing agents.

Method

Twenty-two participants with at least two hypersensitive teeth were randomly selected. Participants were divided into two groups with 22 teeth each, and a desensitizing agent (Fluoraphat Pro and Vivasens) was applied accordingly. Participants were recalled the next day, and a decrease in sensitivity (if any) was evaluated by self-reporting using a 5-point Likert-type scale. Relative Risk (RR) was used to compare the likelihood of sensitivity between two groups and Cohen's d to measure the effect size. A value of p<0.05 was considered significant for all statistical purposes.

Result

The relative risk (RR) results indicated that Fluoraphat Pro has significantly lower RR than Vivasens for patients with dental sensitivity (RR = 0.350, 95% CI 0.187 - 0.654, z = 3.28, p = 0.001). Fluoraphat Pro was significantly more effective concerning lower sensitivity in teeth located in the upper right (dCohen = 3.217, p = 0.038) and lower right (dCohen = 3.193, p = 0.017) of the mouth than VivaSens®.

Conclusion

The two commercially available desensitizing agents Fluoraphat Pro and Vivasens tested in this study were effective in controlling DH. Fluoraphat Pro was more efficient in relieving the risk of dental hypersensitivity than VivaSens®. Further research is needed to evaluate the long-term effects of the desensitizing agents and compare DH reduction with other marketed desensitizing products.

## Introduction

Dentine hypersensitivity (DH) is a phenomenon observed in structurally intact teeth that are devoid of pathological or defective conditions. It is characterized by a short, sharp pain in response to stimuli, typically thermal, evaporative, tactile, osmotic, or chemical, which cannot be attributed to any other dental defect or pathology [1]. DH may cause discomfort for the patient during activities such as eating, drinking, maintaining dental hygiene, and perhaps even breathing. These possible constraints on daily activities are significantly impacting the patient's quality of life. The observed frequency of DH exhibits a wide range, from 4% to 74%, as documented in various studies [2]. The first molars, canines, and premolars of the upper and lower jaws exhibit a higher propensity for susceptibility [3,4].

The pathogenesis of DH is intricately connected to the phenomenon of dentinal tubule exposure. DH can manifest as a result of dental erosion caused by various factors. These include acidogenic diets, vigorous tooth-brushing practices, imprudent utilization of whitening products, gingival recession, periodontal debridement, or surgical interventions [4]. Consuming beverages at low temperatures is the most frequently observed triggering stimulus [5]. Management practice typically encompasses strategies that can effectively accomplish one or both of the following objectives: i) perturbation of the fluid dynamics within the intricate network of dentinal tubules and ii) manipulation or hindrance of the neural signaling within the pulpal nerve pathway [6].

A wide range of products encompassing various functions have been carefully developed after extensive experimentation. These products can fulfill the objectives mentioned above. They can be employed either within a professional setting or within the confines of one's home, depending upon the severity of the condition. These particular products are frequently denoted as desensitizing agents [7]. Grossman postulated that an optimal desensitizing agent ought to possess the attributes of being non-irritating to the dental pulp, exhibiting a relatively painless application process, facilitating ease of administration, manifesting prompt action, demonstrating sustained effectiveness over extended durations, and refraining from inducing tooth discoloration [8]. The utilization of dentine desensitizers has emerged as a common approach to managing DH. Frequently employed desensitizers consist of various constituents, including fluoride, triclosan, benzalkonium chloride, ethylene diamine tetraacetic acid, and glutaraldehyde [9].

DH is predicted to increase in prevalence due to the growing life expectancy of the population and the prevalence of vital or poorly restored teeth, which are more susceptible to tooth wear. Evaluating and comparing the effectiveness of the different treatment modalities for DH is crucial to assisting dental professionals in managing this oral condition. The gold standard treatment approach for DH has not been found until now. The majority of treatment is self-administered by applying toothpaste products; however, professionally applied DH agents are usually reserved for severe non-responding patients. Assessing and comparing the effectiveness of various treatment medications for DH is essential to assisting dental practitioners in managing this condition.

Two well-known agents, Fluoraphat Pro (Neumünster, Germany) and VivaSens® (Ivoclar Vivadent, Schaan, Liechtenstein, Switzerland), have been used as dentine desensitizers and are now widely available for commercial use [10]. However, additional clinical investigations are needed to compare the effectiveness of these agents in reducing DH in patients. Hence, this study evaluated the DH risk reduction after the use of Fluoraphat Pro and VivaSens® in-office desensitizing agents.

## Materials and methods

Research design

**Figure 1:**
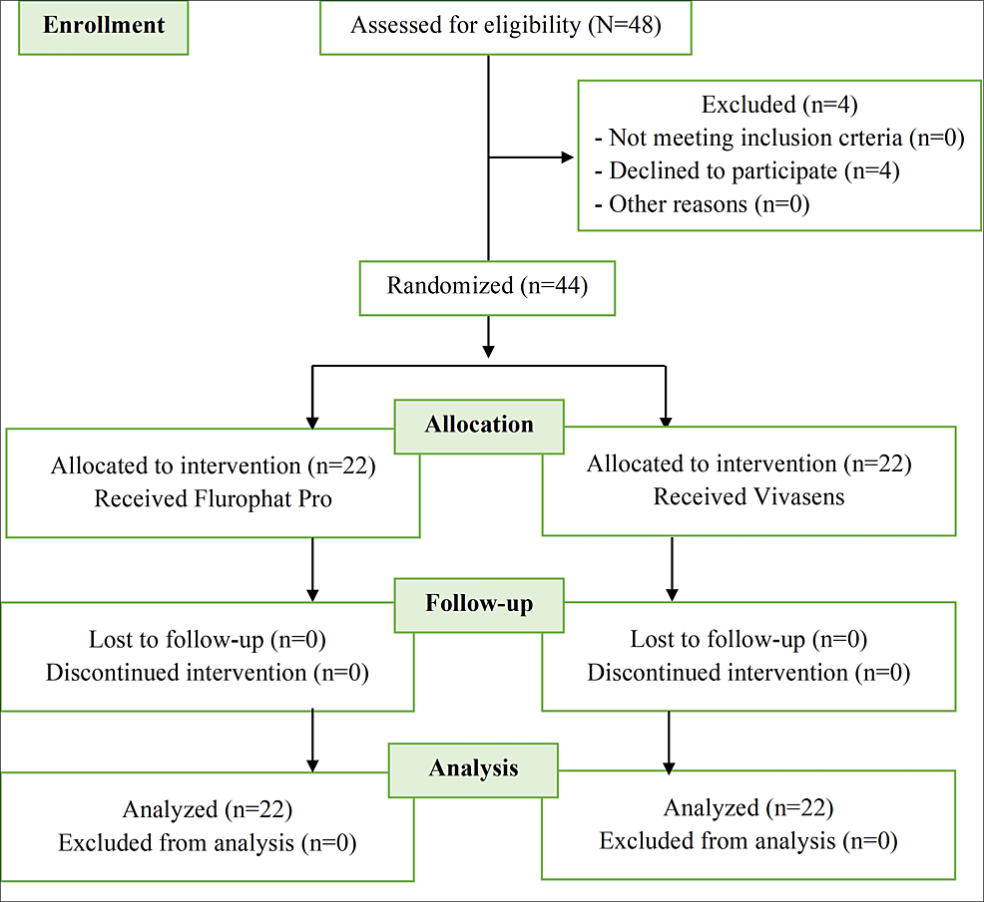
CONSORT flow diagram of the study trial n=number of participants

This was a randomized, parallel-group clinical study that compared the efficacy of Fluoraphat Pro and VivaSens® dentin desensitizing agents in reducing the risk of DH. This study followed the Consolidated Standards of Reporting Trials (CONSORT) guidelines. The flow chart of the trial is shown in Figure 1.

Ethical clearance and informed consent

The study proposal was submitted to the research and innovation center of Riyadh Elm University (REU), and approval for the study (RC/IRB/2019/67) was obtained from the REU ethics committee. The researcher fully explained to the study subjects the risks and benefits of participating in the study. All the research information was provided to the participants. The participants, after understanding the content in their local language, signed an informed consent form on paper. The study was carried out in accordance with the Declaration of Helsinki. Information confidentiality was assured, and the data were utilized for scientific research and publication purposes.

Study participants

Sample Size Calculation

Twenty-two patients (11 males and 11 females) visiting the restorative dentistry and endodontics department of Riyadh Elm University with a history of DH to hot/cold, sweet/sour, or mechanical stimuli on at least two teeth were recruited in this trial. A sample of 44 DH teeth was calculated based on the large effect size, alpha error probability of 0.05, and power of 0.80 with an allocation ratio of 1:1. 

*Data Collection* 

Patients presenting with DH on the buccal surface of the teeth were evaluated by an experienced resident using a probe tip and a 4X magnifying lens. The probe's tip was positioned at a right angle to the tooth's surface and inserted until it reached the bottom of the gingival sulcus, passing the cementoenamel junction. DH was assessed using a five-point self-reported Likert scale ranging from 0 to 4 [11]. Each grade denotes the precise level of severity of the DH: 0 - Absence of sensitivity; 1 - Negligible sensitivity; 2 - Slight sensitivity; 3 - Moderate sensitivity; and 4 - Intense sensitivity. The 44 DH teeth were selected based on the following criteria.

Eligibility criteria

Inclusion Criteria

Both male and female patients aged between 18 and 50 years having a minimum of two teeth displaying inherent DH were selected in this trial. The study teeth were devoid of any iatrogenic factors or detrimental oral practices and had sound tooth structure without any carious lesions. All the teeth exhibiting grade 4 DH on the Likert scale were included in this study.

Exclusion Criteria

DH teeth with gingival recession, dental caries, restoration, fracture. crack, and microleakage were excluded from the study. Participants who did not agree to give informed consent were excluded from the trial.

Desensitizing agents

Two different desensitizing agents, namely, VivaSens and Fluoraphat Pro were used to conduct this experiment.

VivaSens (Ivoclar Vivadent, Liechtenstein, Switzerland)

Composition (weight by %): Varnish (ethanol, water, and hydroxypropyl cellulose) (74.6), polyethylene glycol dimethacrylate, methacrylate (PEG-DMA) (25.0), potassium fluoride (0.3), aroma (0.1), and phosphonic acid methacrylate (4 mg).

Mechanism of action: The main mechanism of action of VivaSens® involves the occlusion of dentinal tubules by the precipitation of calcium ions from the dentin fluid, together with the co-precipitation of PEG-DMA contained in the desensitizer solution. Another mechanism by which VivaSens® operates is by producing salts generated by acid. The dentinal fluid has a high concentration of calcium ions, and the phosphonic acid methacrylate found in VivaSens® leads to the formation of calcium salts with limited solubility. Consequently, these salts precipitate inside the dentinal tubules. The desensitizer contains another acid component known as methacrylate-modified polyacrylic acid, which serves as a complex builder and contributes to the production of additional salts. Potassium ions in the fluoride component play a supporting role in salt precipitation.

Application method of VivaSens: Before application, cotton pellets are used to clean and dry all surfaces of the teeth. Three drops of the VivaSens desensitizing liquid are extracted from the container and dispensed into the mixing block using a squeezing action. The liquid is thoroughly blended using the applicator brush that has been pre-coated. The liquid is administered to the damaged teeth with a gentle rubbing motion, using disposable brushes, for a minimum duration of 10 seconds. This process ensures the equal distribution of the liquid throughout the tooth surface. Subsequently, a delicate stream of air is used to dry the teeth. Patients are advised to abstain from consuming food and beverages or engaging in oral hygiene practices, such as tooth brushing, for 30 minutes after the completion of the treatment. No potential side effects were reported after the application of VivaSens.

Fluoraphat Pro (Promedical Neumunster, Germany)

Fluoraphat is available as a varnish supplied in tubes with disposable micro-brush applicators.

Composition: Ethanol suspension of colophony, artificial flavoring (melon), Xylitol sweetener, and 5% sodium fluoride (1 ml of the material contains 50 mg of 5% sodium fluoride with approximately 22.6 mg fluoride).

Mechanism of action: Occlusion of dentinal tubules and blocking of the dentinal pain pathway.

Application method of Fluoraphat Pro: The teeth surfaces are cleaned of plaque and other debris. Excessive saliva or moisture is removed from the area to be treated for best results. Fluoraphat Pro is applied uniformly as a thin film (0.25 ml to 0.40 ml) over the entire surface of the tooth to be treated. The area is then moistened by either gentle rinsing or by saliva to ensure the proper setting of the varnish. The patient is instructed to avoid brushing and flossing teeth and consuming alcohol and solid foods for the next four hours. No potential side effects were noted after application of Fluoraphat Pro.

All patients subjected to these treatments were recalled after the first day and asked about improved dental sensitivity. They were asked to self-report the improvement in dentinal hypersensitivity using a Likert scale ranging from 0-4 [11]. Each grade expresses the specific type of severity of the DH: 0 - No sensitivity; 1 - Very mild sensitivity; 2 - Mild sensitivity; 3 - Moderate sensitivity; and 4 - Severe sensitivity.

Statistical analysis

Descriptive statistics of percentage, median, and mode values were calculated for the DH scores. Relative risk (RR) values were used to compare the likelihood, or chance, of sensitivity between two materials. Cohen's d was calculated to measure the effect size. Analysis was performed using Statistical Package for Social Sciences (Version 26.0; IBM Corp., Armonk, NY). A value of p<0.05 was considered significant for all statistical purposes.

## Results

A total of 22 participants (11 males and 11 females, aged 18-50 years) suffering from DH were recruited for this study. Most of the study participants were in the age category of 29-39 years (10; 45.5%), followed by 40-50 years (8; 36.4%), and 18-28 years (4; 18.2%). The educational level showed that many had a college level of education (17; 77.3%), followed by secondary school (5; 22.7%). A large proportion of participants were employed in the government sector (10; 45.5%), followed by the private sector (4; 18.2%), students (4; 18.2%), and homemakers (4; 18.2%).

The median score of VivaSens® (1) was higher than Fluoraphat Pro (0) by one unit, which indicates that more patients chose "No sensitivity" for Fluoraphat Pro (68.2%) than for VivaSens® (9.1%). Nearly 59.1% of patients reported very mild sensitivity after applying VivaSens® (Table 1).

**Table 1:** Relative risk (RR) along with the 95% confidence interval and z-test results

Location	RR	95% CI		Z Score	P Value
Upper Right	0.127	0.009	1.816	1.520	0.128
Upper Left	0.500	0.250	0.990	1.961	0.049
Lower Left	0.600	0.290	1.220	1.399	0.162
Lower Right	0.106	0.070	1.550	1.640	0.101
OVERALL	0.350	0.187	0.654	3.280	0.001

**Figure 2:**
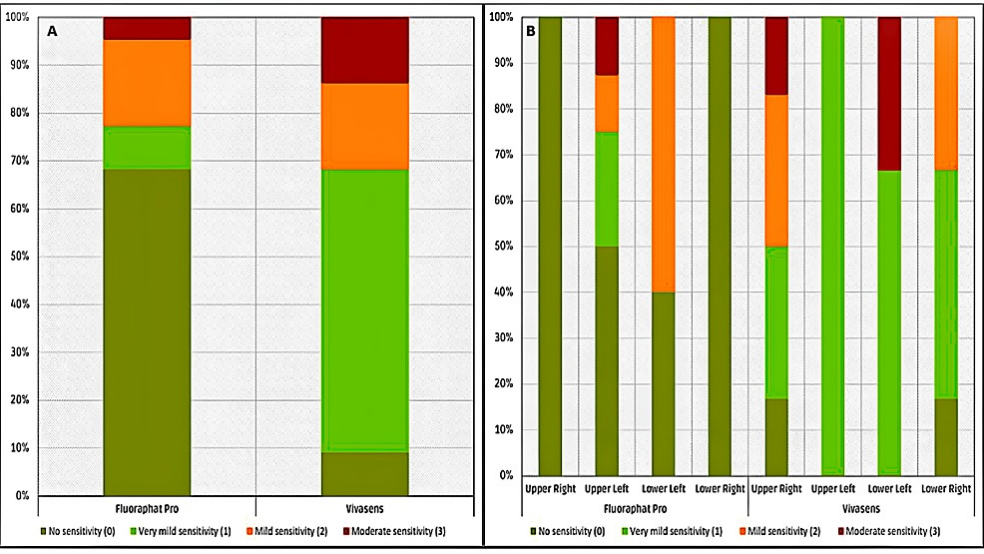
Bar plot illustrating the: A. Percentage of different levels of sensitivity by material. and B. Percentage of different levels of sensitivity by tooth location and material

It is evident in Figure 2 that on the teeth to which Fluoraphat Pro was applied, there were 68.2% more cases with "No sensitivity" than those with VivaSens® (9.1%).

**Figure 3:**
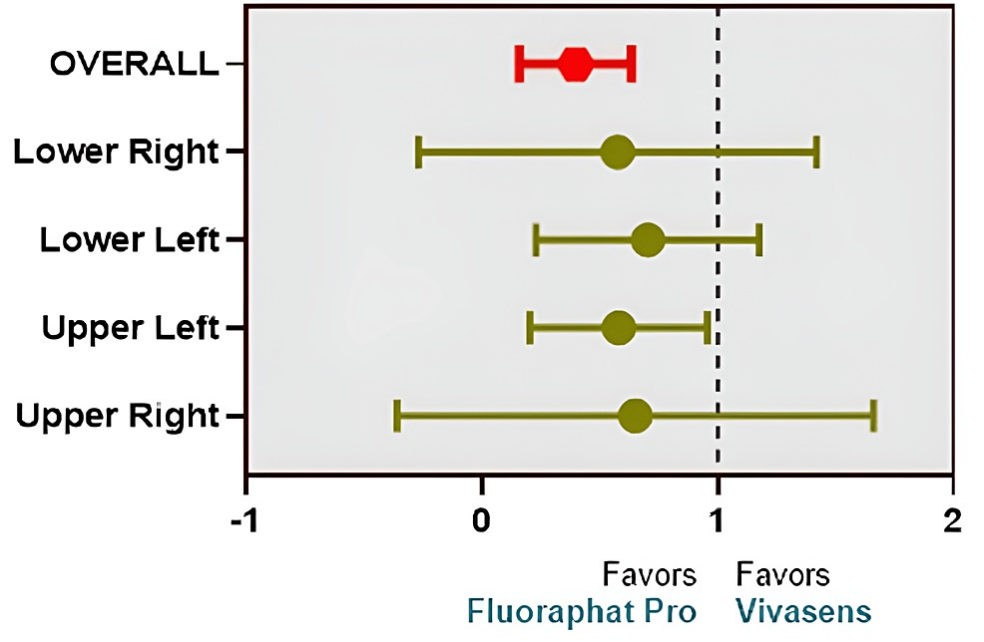
Relative risk (RR) forest plot

The RR results indicate that Fluoraphat Pro has significantly lower RR than Vivasens for patients with dental sensitivity (RR = 0.350, 95% CI 0.187 - 0.654, z = 3.28, p = 0.001). In addition, different teeth locations showed different RR values when comparing the efficiency of the two materials. Statistically, no significant sensitivity difference was observed between the two materials in teeth located in the upper right (RR = 0.127, 95% CI 0.009 - 1.816, z = 1.520, p = 0.128), lower left (RR = 0.600, 95% CI 0.290 - 1.220, z = 1.399, p = 0.162), and lower right (RR = 0.106, 95% CI 0.070 - 1.550, z = 1.640, p = 0.101). However, there has been a significant efficiency difference between Vivasens and Fluoraphat Pro in the teeth located in the upper left, namely, lower risk (RR = 0.500, 95% CI 0.250 - 0.990, z = 1.961, p = 0.049). The results of relative risk are illustrated in Table 2 and Figure 3.

**Table 2:** Effect sizes Eta squared and Cohen's d along with the Wilcoxon rank test

Tooth Location	Eta Squared	Cohen's d	p	Wilcoxon
Upper Right	0.720	3.217	0.038	12.0
Upper Left	0.662	2.802	0.459	48.0
Lower Left	0.662	2.802	0.549	27.0
Lower Right	0.718	3.193	0.017	17.5
OVERALL	0.185	0.952	0.003	373.5

**Figure 4:**
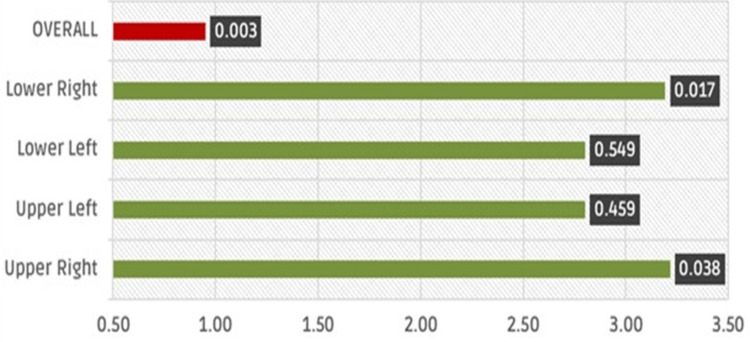
Effect size (Cohen's d) and statistical significance

According to the results in Table 2, the effect sizes are all in the "large effect" range. By performing the Wilcoxon test, these large effects were examined. The results indicated that Fluoraphat Pro is significantly more effective concerning lower sensitivity in teeth located in the upper right (dCohen = 3.217, p = 0.038) and lower right (dCohen = 3.193, p = 0.017) of mouth than VivaSens®. The results also demonstrated a better efficiency for Fluoraphat Pro since dCohen = 0.952 with a p-value of 0.003. The effect size (dCohen) of Fluoraphat Pro compared to VivaSens® is illustrated in Figure 4, along with the p values.

## Discussion

Several studies have shown that DH is a growing dental condition in Saudi Arabia and affects a significant population in the kingdom. Hamasha et al. (2019) concluded that 33% of the study sample suffered from DH with sensitivity in an average of 4.8 teeth per person [12]. Similarly, a multinational study conducted across six Arab countries observed relatively high levels of dentine hypersensitivity (30% across all participants) and dentine exposure (45.5%) [13]. These studies indicate that within the region where this research was conducted, the prevalence of DH is on the rise, and there is a need for more research on effective management strategies and products in the treatment of DH. To the best of our knowledge, this is the first study that compares Fluoraphat Pro and VivaSens® desensitizing agents and presents insight into choosing an effective treatment option for DH.

In our study, VivaSens ® has proven to have good efficacy while other studies that compared the efficacy of VivaSens® with other desensitizing agents have given mixed results. Some reported superior performance of VivaSens® than other agents, while others reported no difference in efficacy between different agents. Pamir et al. (2007) evaluated three desensitizing agents: Seal&Protect (Dentsply DeTrey GmbH, Konstanz, Germany), Vivasens, and BisBlock (BISCO, Schaumburg, IL, USA) and used distilled water as a placebo. The subjects were recalled after four weeks of material application, and it was observed that there was no significant difference in the alleviating effects of the three agents, and all three performed well [14].

Furthermore, our study results are in complete agreement with an in-vitro study conducted by Pathan et al. (2016) that evaluated dentinal tubule occlusion by three desensitizing agents, i.e. VivaSens, Admira Protect (VOCO America Inc., Indian land, South Carolina, USA), and Neo Active Apatite suspension (nanohydroxyapatite) (Ghimas, Bologna, Italy). All three desensitizers showed some potential for occlusion of the tubules, but Admira Protect and the least Neo Active Apatite suspension showed the best efficacy. In contrast, VivaSens® showed efficacy somewhere between the two, establishing it as an acceptable desensitizing agent [15].

Our study finding is similar to another study that compared the desensitizing capacity of VivaSens® and diode laser in the treatment of DH and observed that both methods were equally effective in the occlusion of dentinal tubules, and no significant difference was observed between the two groups [16]. Moreover, our finding is in line with a more recent study conducted by Mushtaq et al. in 2019, that used scanning electron microscopy to evaluate the occluding ability of three desensitizing agents: Gluma desensitizer® (Kulzer), VivaSens® (Ivoclar Vivadent) and MS Coat® (Sun Medical). This study concluded that MS Coat demonstrated superior results in dentinal tubule closure, followed by VivaSens and Gluma desensitizer [17].

On the other hand, Fluoraphat Pro is the other desensitizing agent of this present research and has demonstrated results superior to VivaSens in this study. Fluoraphat Pro is a desensitizing varnish containing 5% sodium fluoride. A study conducted in 2012 revealed that the desensitizer containing sodium fluoride performed significantly better than the other at one- and three-month intervals [18]. Another study that employed 2% sodium fluoride iontophoresis displayed a significantly better reduction in DH than the one treated with the HEMA-G solution at one- and three-month intervals [19].

Madhavan et al. evaluated the efficacy of propolis, sodium fluoride, CPP-ACP F, and placebo in treating DH. They observed that all the materials used effectively reduced DH, although the rapidity of action over the observed three-month period differed. Propolis was the most efficient in treating DH, casein phosphopeptide-amorphous calcium phosphate (CPP-ACP) F was the least efficient, and sodium fluoride performed moderately well in reducing DH [20]. These studies demonstrate that sodium fluoride is a desensitizing agent that has shown consistently good results in alleviating dentinal hypersensitivity. It is often a better agent than its commercial and in-office counterparts in treating dentinal hypersensitivity. These findings concur with the results of this research where Fluoraphat Pro, which contains 5% sodium fluoride, has been shown to perform better than VivaSens®, which also contains fluoride but in the form of potassium fluoride.

One possibility for this difference in performance may be the difference in fluoride concentration in the two formulations. VivaSens® contains only 0.3% potassium fluoride while Fluoraphat Pro contains 5% sodium fluoride, and in a 1 ml dispensation, offers 50 mg of sodium fluoride, which provides roughly 22.6 mg of fluoride. Another advantage that Fluoraphat Pro seems to have over VivaSens® is that the former can adhere to wet surfaces and is tolerant to moisture and saliva while the latter is sensitive to moisture, which requires proper drying of teeth surfaces before application. This moisture sensitivity may also lead to loss of material after the application of VivaSens® and problems with prolonged adhesion due to saliva in the mouth. In contrast, Fluoraphat Pro, tolerant to moisture, will adhere to moist tooth surfaces for more protracted periods after application, increasing contact time and enhancing the material's efficacy.

The upper and lower quadrants on the right side were more responsive to Fluorophat Pro treatment than the left. Differences in the performance of the same material under the same conditions can occur due to differences in the film thickness of the applied desensitizing agent and the influence of gravity [21]. The factors that affected the difference in our study could not be entirely ascertained. Since all teeth on one side demonstrated better results than the other in this study, it can be assumed that chairside conditions like the inclination of the head of patients due to the manner of application by the operator may be responsible for better application of the product on one side than the other. This study was subject to some limitations which need to be considered. The small sample size for a within-subject design caused the power of our analysis to decrease, and the probability of Type I and II errors increased. By using RR and effect size, it was tried to cover this limitation and illustrate descriptively and, to some extent, inferentially how the efficiency of these two materials could differ.

## Conclusions

The study findings suggest that Fluoraphat Pro and VivaSens desensitizing agents tested in this investigation were effective in controlling DH. Fluoraphat Pro (68.2%) showed better results in alleviating DH than VivaSens (9.1%). Further investigations are needed to assess the long-term effects of the Fluoraphat Pro and VivaSens desensitizing agents and to compare the DH reduction with that of alternative desensitizing agents.
